# A Potentially Catastrophic Anatomical Variation: Aberrant Internal Carotid Artery in the Middle Ear Cavity

**DOI:** 10.1155/2013/743021

**Published:** 2013-04-02

**Authors:** Togay Muderris, Sami Bercin, Ergun Sevil, Huseyin Cetin, Muzaffer Kiris

**Affiliations:** ^1^Department of Otorhinolaryngology-Head and Neck Surgery, Ataturk Training and Research Hospital, Bilkent, 06800 Ankara, Turkey; ^2^Department of Otorhinolaryngology-Head and Neck Surgery, Yıldırım Beyazıt University Medical Faculty, Ankara, Turkey; ^3^Department of Radiology, Ataturk Training and Research Hospital, Ankara, Turkey

## Abstract

Aberrant internal carotid artery (ICA) is a rare but a very important vascular anomaly of temporal bone. Misdiagnosis of the anomaly may lead to massive hemorrhage and severe complications during otologic procedures. It is essential to keep this anomaly in mind for any otologic surgeon to prevent catastrophic complications. We present a case of aberrant ICA appeared as a nonpulsatile middle ear mass. The patient had a complaint of hearing loss, and the otoscopic examination of the patient revealed a tympanic membrane perforation and a blue-reddish retrotympanic mass. Multidetector computed tomography (MDCT) is a useful tool that may provide excellent visualization of temporal bone for the diagnosis of aberrant ICA. Otolaryngologists should be aware of the possibility of a vascular anomaly of temporal bone when a patient presents with a blue-reddish mass in the middle ear.

## 1. Introduction

 An aberrant internal carotid artery (ICA) in the middle ear is a rare but an important vascular anomaly of the temporal bone that every otolaryngologist and radiologist should know about [1]. It is generally accepted to be a collateral pathway that occurs as a result of agenesis of the first embryonic segment of the ICA [2]. The clinical symptoms and signs of aberrant ICA are often nonspecific and include hearing loss, pulsatile tinnitus, and a retrotympanic mass behind the anteroinferior part of the membrane [3, 4].

It can mimic glomus tumors and other vascular temporal bone lesions (dehiscent jugular bulb, cholesterol granuloma and petrous carotid aneurysms, and pseudoaneurysms and hemangiomas) [5, 6]. A misdiagnosis of this anomaly may subject patients to aural bleeding during myringotomy or tympanotomy and serious consequences which is potentially life threatening.

We report a case to describe and highlight the clinical and radiological features of the aberrant internal carotid artery and to discuss strategies for diagnosis and management.

## 2. Case Report

**Figure 1:**
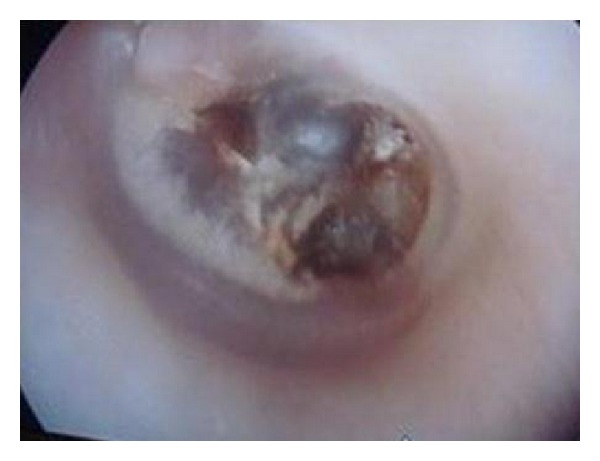
Nonpulsatile blue-reddish mass behind the perforated tympanic membrane.

A 47-year-old female patient presented to our clinic with a five-year history of hearing loss in the left ear. Her otoscopic examination revealed a central tympanic membrane perforation and a nonpulsatile blue-reddish mass protruding from the middle ear cavity. She did not have a history of discharge or bleeding from her left ear (Figure 1). Pure tone audiogram showed 38 dB conductive hearing loss of the left ear.

**Figure 2:**
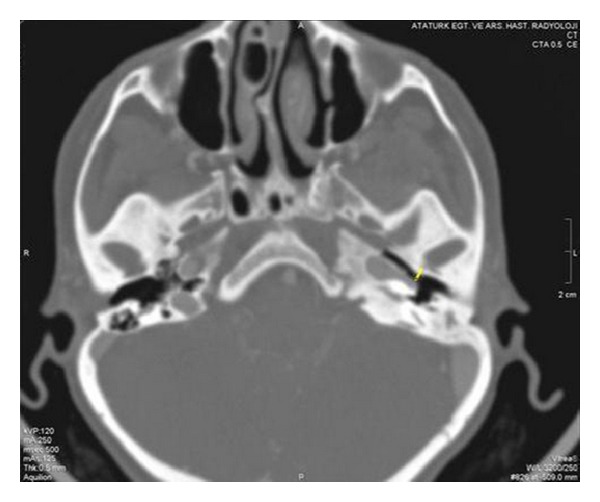
Axial CT scan of the temporal bone showing the aberrant carotid artery, entering the tympanic cavity through a dehiscent carotid plate.

**Figure 3:**
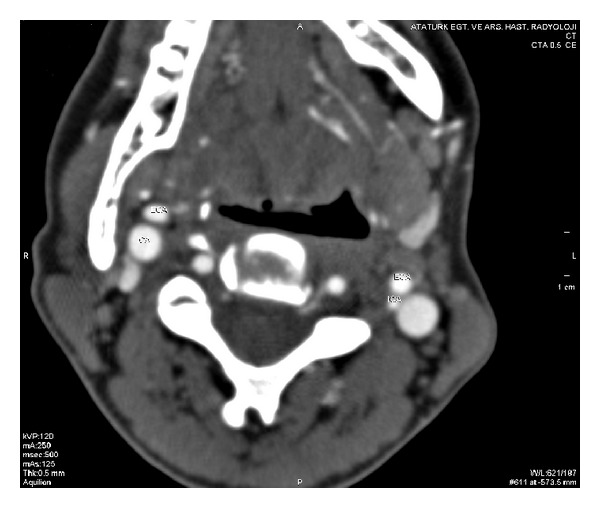
MDCT showing reduced diameter and lateralization of the left ICA compared to the right ICA.

Computerized tomography scanning of the temporal bone was performed, and a left-sided aberrant ICA with bony dehiscence of the carotid canal was seen (Figure 2). Multidetector computed tomography (MDCT) of the temporal bone revealed protrusion of the internal carotid artery into the middle ear and showed a reduced diameter and lateralization of the left ICA compared to the right ICA (Figure 3).

**Figure 4:**
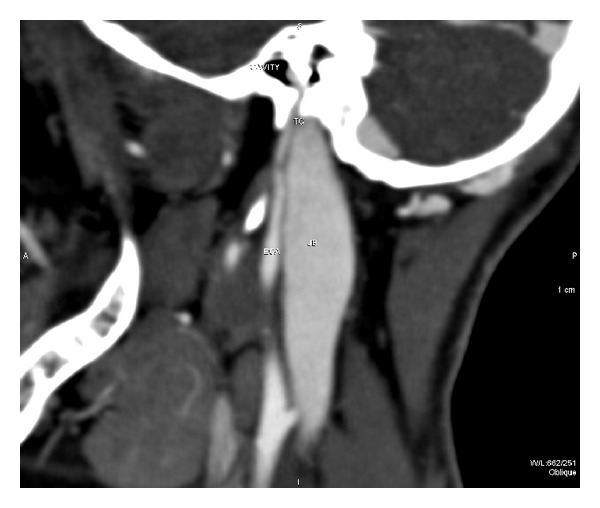
MDCT showing that the ECA was entering to the left middle ear, and the tympanic canaliculus was seen as expanded and had a connection with the horizontal (petrous) part of internal carotid artery. JB: jugular bulb, ECA: external carotid artery, and TC: tympanic canaliculus.

 On the MDCT, the tympanic canalicus was seen as expanded, and the external carotid artery (ECA) was entering to the left middle ear and had a connection with the horizontal (petrous) part of the internal carotid artery (Figure 4). 

The right ICA was normal, and there was no intracranial aneurysm, arteriovenous malformation, or other persistent embryological vessels.

She was informed about the diagnosis and the possible complications of middle ear interventions. The patient was managed with conservative treatment.

## 3. Discussion 

Vascular anomalies of the temporal bone are rare, but they may become very important during middle ear surgery. Possible vascular variations of the temporal bone are the aberrant internal carotid artery, high jugular bulb, persistent stapedial artery, dehiscent carotid artery canal, and dehiscent high jugular bulb [4, 7]. An aberrant ICA has an incidence of less than one percent [7, 8].

Moret at al. explained that the aberrant ICA actually represents an enlarged inferior tympanic artery anastomosing with an enlarged caroticotympanic artery when the cervical segment of the ICA is underdeveloped or has regressed during embryogenesis. The aberrant ICA enters the tympanic cavity through an enlarged inferior tympanic canaliculus, courses through the tympanic cavity under the cochlear promontory anteriorly, and then medially enters the horizontal carotid canal through a dehiscence in the carotid plate [9]. As distinct from previous reports, our case showed us actually that ascending pharyngeal artery does not enter the tympanic canaliculus, and external carotid artery enters itself. Rarely, the carotid plate may be dehiscent allowing the artery to herniate into the tympanic cavity [10].

One of the most accepted theories of aberrant ICA belongs to Lasjaunias and Santoyo-Vazquez [11]. According to this theory, the persistence of embryologic pharyngeal artery causes the blood flow from ascending pharyngeal artery to inferior tympanic artery, and this will delay the progress of the cervical part of ICA. 

There are specific radiological and clinical findings of aberrant ICA: (1) soft tissue mass in the middle ear; (2) defective thin bonny membrane around the mass; (3) the mass lies from the promontorium to the tympanic membrane; (4) the artery which lies below the incudostapedial joint causes conductive type hearing loss; (5) the absence of the proximal part of the carotid canal; (6) the enlargement of tympanic annulus [1]. 

The clinical symptoms and signs of an aberrant ICA are often nonspecific or absent. Hearing loss is the most common presenting symptom; others include pulsatile tinnitus, serous otitis media, otalgia, and aural fullness [3, 6, 8]. In this case, the only symptoms were conductive hearing loss and aural fullness. Audiometric results and aural fullness may be attributed to malleus or incus blockage or associated middle ear disease. The patient's clinical situation could have been considered as a glomus tumor or other vascular malformation. However, a tympanic mass due to an aberrant ICA looks different from a glomus tumor: anterior, pulsatile, and white or rosy, but it is not always pulsatile such in this case. After a careful assessment of the patient, if the surgeon has any clinical doubt about the possibility of an aberrant ICA, a CT scan of the temporal bone should be performed before any middle ear surgery [5, 12].

Radiological investigations should be performed for evaluation of middle ear vascular anomalies [3, 6]. Computed tomographic scan has become the standard for diagnosis. It is useful to determine the dehiscence of the bony canal of the ICA and the relation between the ICA and middle ear structures [13]. MDCT provides excellent visualization of the external carotid artery anastomoses with the internal carotid artery in the middle ear cavity. In this case, MDCT showed the dehiscent bony plate along the petrous part of the ICA, a reduced diameter of the petrous ICA, hypoplasia of the vertical segment of the carotid canal, an external carotid artery entered the tympanic canaliculus itself, any brunch of carotid artery did not enter, and an enhancing mass in the hypotympanum. On MDCT scan, these were the main parameters that we used to diagnose the aberrant ICA. 

In case of an asymptomatic and proven aberrant ICA, most authors recommend a conservative approach [14, 15], as opposed to Ruggles and Reed [16], who advocated surgery to relieve the patient of troublesome symptoms (tinnitus and hearing loss) and to prevent possible destruction of the middle structures and formation of an aneurysm. In case of chronic middle ear infection or to reduce tinnitus, however, surgical treatment is indicated, including covering the aberrant vessel with bone and soft tissues or trimming the handle that touches the exposed ICA [16, 17]. Therefore, the benefit of the surgery must be debated against the risk of possible consecutive neurological disorders and serious bleeding complications.

In conclusion, the otolaryngologist should be aware of the vascular malformation of the temporal bone. We thus recommend that aberrant ICA should be kept in mind in otologic patients to prevent possible dramatic surgical complications. The diagnosis of AICA is very easy with the help of MDCT. Also this is the first report to show the aberrant internal carotid artery different from the other reports. 

## References

[1] Caldas JGMP, Iffenecker C, Attal P, Lasjaunias P, Doyon D (1998). Anomalous vessel in the middle ear: the role of CT and MR angiography. *Neuroradiology*.

[2] Roll JD, Urban MA, Larson TC, Gailloud P, Jacob P, Harnsberger HR (2003). Bilateral aberrant internal carotid arteries with bilateral persistent stapedial arteries and bilateral duplicated internal carotid arteries. *American Journal of Neuroradiology*.

[3] Windfuhr JP (2004). Aberrant internal carotid artery in the middle ear. *The Annals of Otology, Rhinology & Laryngology. Supplement*.

[4] Eryilmaz A, Dagli M, Cayonu M, Dursun E, Gocer C (2008). An aberrant internal carotid artery in the temporal bone presenting as a middle-ear mass: a case report. *Journal of Laryngology and Otology*.

[5] Endo K, Maruyama Y, Tsukatani T, Furukawa M (2006). Aberrant internal carotid artery as a cause of objective pulsatile tinnitus. *Auris Nasus Larynx*.

[6] Sauvaget E, Paris J, Kici S (2006). Aberrant internal carotid artery in the temporal bone imaging findings and management. *Archives of Otolaryngology*.

[7] Koesling S, Kunkel P, Schul T (2005). Vascular anomalies, sutures and small canals of the temporal bone on axial CT. *European Journal of Radiology*.

[8] Botma M, Kell RA, Bhattacharya J, Crowther JA (2000). Aberrant internal carotid artery in the middle-ear space. *Journal of Laryngology and Otology*.

[9] Moret J, Delvert JC, Bretonneau CH, Lasjaunias P, de Bicêtre CH (1982). Vascularization of the ear: normal-variations-glomus tumors. *Journal of Neuroradiology*.

[10] Myerson MD, Ruben H, Gilbert JG (1934). Anatomic studies of the petrous of the temporal bone. *Archives of Otolaryngology*.

[11] Lasjaunias P, Santoyo-Vazquez A (1984). Segmental agenesis of the internal carotid artery; Angiographic aspects with embryological discussion. *Anatomia Clinica*.

[12] Aladwan A, Mack M, Gstöttner W, Vogl TJ (2005). Duplication of internal carotid artery: a rare case of tympanic mass. *European Radiology*.

[13] Yoshida M, Karino S, Yamasoba T (2007). Aberrant internal carotid artery protruding through a tympanic membrane perforation. *Otolaryngology*.

[14] Duclos JY, Darrouzet V, Martel J, Berge J, Calas V, Bebear JP (2000). Aberrant internal carotid artery in the middle ear. Case report. *Revue de Laryngologie Otologie Rhinologie*.

[15] Cole RD, May JS (1994). Aberrant internal carotid artery. *Southern Medical Journal*.

[16] Ruggles RL, Reed RC (1972). Treatment of aberrant carotid arteries in the middle ear: a report of two cases. *Laryngoscope*.

[17] Glasscock ME, Dickins JR, Jackson CG (1980). Vascular anomalies of the middle ear. *Laryngoscope*.

